# ZFP36L2 Is a Potential Prognostic Marker of IL1β^+^ Osteosarcoma †

**DOI:** 10.3390/biomedicines12122861

**Published:** 2024-12-17

**Authors:** Peiyao Hao, Piaopiao Luo, Shenglin Xu, Zhenhua Ren, Hong Zhao, Xiang Nan

**Affiliations:** 1 Department of Anatomy, School of Basic Medicine, Anhui Medical University, Hefei 230032, China; hpy60@outlook.com (P.H.); 17883983809@163.com (P.L.); ZH.R132@outlook.com (Z.R.); 2 Department of Orthopedics, The First Affiliated Hospital of Anhui Medical University, Hefei 230022, China; leaf1842@outlook.com; 3 Department of Systems Medicine and Bioengineering, T. T. and W. F. Chao Center for BRAIN, Houston Methodist Neal Cancer Center, Houston Methodist Hospital, Weill Cornell Medicine, Houston, TX 77030, USA

**Keywords:** osteosarcoma, ZFP36L2, IL1β

## Abstract

**Background:** Osteosarcoma stands as the predominant bone malignancy afflicting children and young adults. Despite strides in treatment, the enduring reality is that the long-term survival rates for patients grappling with recurrences and metastases linger at a mere 30%. This underscores the pressing demand for novel prognostic markers and therapeutic avenues to improve outcomes and offer hope to those battling this formidable disease. ZFP36L2, a member of the tristetraprolin family of CCCH zinc finger proteins, stands out for its pivotal role in posttranscriptional modifications and its ability to modify tumor microenvironments. **Methods:** We obtained RNA-seq data from TCGA and GTEx cohorts to investigate the expression of ZFP36L2 in tumor and normal tissues. We also utilized GO/KEGG analysis and immune infiltration analysis to verify the relationship between ZFP36L2 and immune cells. A Kaplan–Meier survival curve was used to study the relationship between ZFP36L2 and IL1β in osteosarcoma. Single-cell data analysis and cell–cell communication analysis were used to discover the therapeutic potential of ZFP36L2 in osteosarcoma. **Results:** This study elucidates the specific expression pattern of ZFP36L2 in tumors. ZFP36L2 is associated with metastasis in sarcoma, but has no statistically significant correlation with survival rate. ZFP36L2 has been shown to be associated with T cells and macrophages in the tumor microenvironment through GO/KEGG analysis and immune infiltration analysis. The survival analysis results show that ZFP36L2 can serve as a biomarker in IL1β^+^ osteosarcoma. Single-cell sequencing analysis shows that ZFP36L2 is present in IL1β^+^ macrophages. Cell–cell communication analysis indicates that ZFP36L2 targets TNF in IL1β^+^ osteosarcoma, thereby improving prognosis. **Conclusions:** ZFP36L2 has potential as a prognostic marker in IL1β^+^ osteosarcoma patients.

## 1. Introduction

Osteosarcoma [OS] is the most common malignant bone tumor, mainly affecting children and young adults [1,2]. The unsatisfactory long-term survival of patients with recurrence and metastasis represents a considerable challenge in OS treatment, and only ~30% of these patients survive beyond five years. Moreover, the complex tumor microenvironment in OS hinders the development of novel therapy strategies. Precision medicine can tailor disease treatment plans based on individual differences among patients. It has offered promising prospects for the treatment of advanced cancers, but extremely few achievements have been reported in metastatic OS treatment. Thus, prognostic markers are essential to the enhancement of overall survival and research on the intricate mechanisms underlying aggressive tumor metastasis.

ZFP36L2, which is a member of the tristetraprolin [TTP] family of CCCH zinc finger proteins, exerts its influence through posttranscriptional modifications [3]. The TTP family consists of ZFP36, ZFP36L1, and ZFP36L2, though rodents also possess a fourth family member, ZFP36L3, which is only expressed in the placenta and yolk sac [4]. It is a small group of mRNA binding and unstable proteins that are expressed in almost all eukaryotes [5] and can bind to adenine–uridine-rich elements located in the 3′-untranslated region of mRNAs, thus inducing mRNA decay [3,6]. ZFP36L2 plays a crucial role in macrophage recruitment in the presence of HIF-1α [7], and the complexes between ZFP36L2 and adenine–uridine-rich elements can cause cell cycle arrest [8,9]. In addition, ZFP36L2 is highly correlated with tumor progression, particularly in acute myelocytic leukemia [10,11], lower-grade gliomas [12], and gastric cancer [13]. However, its prognostic power and mechanisms contributing to cancer progression in OS remain unclear.

The tumor microenvironment [TME] plays a pivotal role in tumor metastasis and the response of tumors to therapeutics [14], thereby systemically influencing patients’ survival. Hypoxia is the most prominent impediment in solid tumor treatment because it can disrupt the entire TME [15], promote tumor progression [16], and recruit and retain tumor-associated macrophages [17,18]. Thus, it can lead to poor outcomes. Moreover, it is associated with increased M1 macrophage markers in OS [19,20], such as IL1β [21], and it drives M2 polarization. A hypoxic environment contains IL1β^+^ macrophages that can induce pathogenic inflammation [22]. TME regulation not only affects macrophages, but also other cellular components, hampering T cells’ proliferation and weakening their ability to exert cytotoxic effects [23]. IL1β has been identified as a prognostic marker in multiple types of cancers [24,25], but we found that it failed to act as such in OS patients.

This study [26] is based on our finding that ZFP36L2 overexpression is observed in sarcoma patients. However, its expression is lower in metastatic cases compared to non-metastatic ones. The Kaplan–Meier plot we performed indicates that ZFP36L2 cannot serve as an independent prognostic marker. Given that ZFP36L2 is a posttranscriptional regulator, we hypothesized that it plays an important role in tumor progression and is a potential biomarker. Specifically, we found that ZFP36L2 can be used as a prognostic marker for IL1β-high OS and has potential use in personalized medicine. To further explore its potential mechanism, we performed multiple immune infiltration analyses. Through methods such as QUANTISEQ, TIMER, and CIBERSORT, the results indicate that ZFP36L2 is closely related to macrophages and T cells. Through OS single-cell RNA sequencing, we unveiled the heterogeneous expression patterns of ZFP36L2. Furthermore, cell–cell interaction analysis revealed their intricate interactions with tumor cells, macrophages, and T cells, and their contribution to TNF-induced apoptosis. We explored the prognostic potential of ZFP36L2 and its role in IL1β-high OS, and shed a light on its clinical applications in OS treatment.

## 2. Materials and Methods

### 2.1. Cell Lines and Cell Culture

Cell lines 143B and MNNG/HOS CL, procured from ZQXZbio [Shanghai, China], were authenticated through STR analysis and maintained in a controlled environment with 5% CO_2_ at 37 °C. Then, the cell lines were cultured in DMEM [Hyclone, Logan, UT, USA] supplemented with 10% fetal bovine serum [Gibco, Grand Island, NY, USA] and 1% penicillin–streptomycin solution [Invitrogen, Carlsbad, CA, USA]. We moved 1 × 10^5^ cells into a 60 mm cell culture dish, and after 24 h, the cells occupied approximately 90% of the well plate area. We placed the cells under normoxic and hypoxic conditions, respectively. The normoxic conditions were 37 °C and 21% O_2_, while the hypoxic conditions were achieved by using a dedicated chamber from Billups Rothenberg [San Diego, CA, USA] and exposing cells to 0.1% oxygen for 24 h.

### 2.2. Total RNA Extraction and Quantitative Real-Time PCR [qPCR]

Total RNA was extracted from OS cells with a SparkJade RNA extraction kit [Jinan, China] in accordance with the manufacturer’s protocols. After RNA extraction, RNA reverse transcription was performed with Prime Script RT Master Mix [SparkJade, Jinan, China]. Quantitative real-time PCR [qPCR] was conducted using 2× SYBR Green qPCR Mix with ROX [SparkJade, Jinan, China] and specific primer sequences. The primer sequences were as follows: β-actin: 5′-TCATCACCATTGGCAATGAG-3′ and 5′-CACTGTGTTGGCGTACAGGT-3′; ZFP36L2: 5′-CTGCTGCTGACTGCGGTA-3′ and 5′-ATCCAGACCCACAACTTTGC-3′.

### 2.3. Study Cohorts

Our study leveraged the collective power of four prominent public databases: The Cancer Genome Atlas [TCGA] database, the Genotype-Tissue Expression [GTEx] dataset, the Gene Expression Omnibus [GEO] database, and the TARGET database. For our investigation, we integrated RNA sequencing data obtained from sarcoma samples collected from tumor and normal tissues obtained from the TCGA and GTEx databases. We referenced the SARC dataset, which comprises data from 264 patients, for bioinformatics analysis. The GEO database provides access to GSE162454 and GSE152048 data for single-cell sequencing and cell–cell communication analyses, respectively. Lastly, the TARGET database is a specialized resource for identifying genes and mutations associated with childhood cancer. We use it for research related to osteosarcoma.

### 2.4. Bulk RNA-Seq Data and Processing

We initially converted RNA-seq data obtained from the TCGA and GTEx cohorts into the transcripts per million [TPM] format to streamline subsequent analysis. The TPM data were then processed and transformed into the log2 format. For comprehensive pan-cancer analysis, we employed Gene Expression Profiling Interactive Analysis [GEPIA 2.0], which is a recently developed interactive web server designed for analyzing RNA sequences and expression data from 9736 tumors and 8587 normal samples sourced from the TCGA and GTEx databases, utilizing a standardized processing pipeline. Moreover, we compared the transcription expression levels of cancers and normal tissues by using GEPIA 2.0. Immune infiltration analysis was conducted using the SangerBox [http://sangerbox.com/] [27] pipeline with the default parameters.

### 2.5. Single-Cell Data Analysis

The single-cell sequencing data were obtained from two OS datasets [GSE162454 and GSE152048] in the GEO database. The read10x function was used in the processing of the Seurat object of the single-sample gene expression data. The Seurat software package [version 4.3.1] was used for data quality control, and the Merge function was applied to combine the two OS datasets after batch effect removal with the Harmony package. These procedures ensured consistency across cell data. Subsequently, the FindClusters function from the Seurat software package [resolution = 0.5] was used for primary cell clustering analysis, and visual clustering results were obtained through umap dimensionality reduction analysis. Cell clusters were initially labeled using the single R built-in reference dataset [Blueprint Encode Data], and nine cell subpopulations were identified. The cell subpopulations were annotated on the basis of cell markers in the literature [10]. For instance, subsets within the macrophages group were distinguished, including OCs cells [ACP5 and CTSK], IL1β^+^ macrophages [IL1β], and macrophages. Similarly, subsets representing OS cells [ALPL, RUNX2, and IBSP] and fibroblasts were identified in accordance with gene expression patterns. Finally, eleven distinct cell subpopulations were characterized.

### 2.6. Cell–Cell Communication Analysis

To explore the intricacies of intercellular interactions, we employed CellChat to investigate communication among OS cells, CD8^+^ T cells, and IL1β^+^ macrophages. Initially, annotated single-cell RNA sequencing data were inputted into CellChatDB, which is a tool for analyzing cell–cell communication based on ligands and receptors that is commonly utilized in ligand–receptor studies for single-cell sequencing. Subsequently, leveraging the differential expression of ligands and receptors across distinct cell groups, we identified and quantified interactions [*p* < 0.05] between the cell populations. To further elucidate these interactions, we compared the communication probabilities of ligand–receptor pairs regulated by specific cell populations with each other, utilizing parameter comparisons within the “netVisual_bubble” function. Finally, data with notable biological significance were used for exploring intercellular dynamics within the studied microenvironment.

## 3. Results

### 3.1. ZFP36L2 Is Associated with Metastasis in Sarcomas

**Figure 1:**
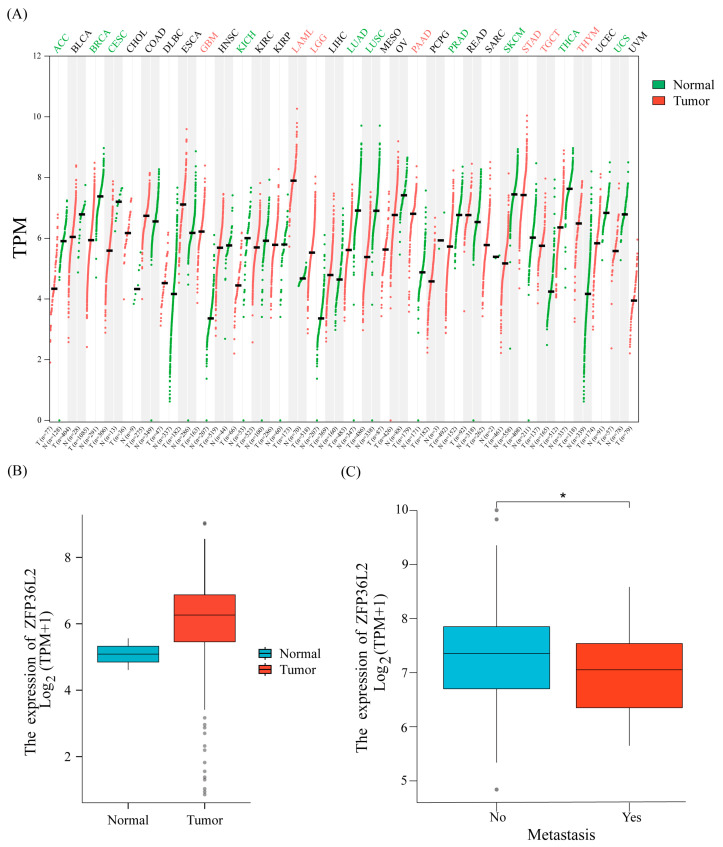
The expression of ZFP36L2 varies across tumor types pan-cancer, with higher expression in sarcoma compared to normal tissue and lower expression in metastatic sarcoma than in non-metastasis. (**A**) ZFP36L2 expression pan-cancer. (**B**) ZFP36L2 expression in sarcoma. (**C**) ZFP36L2 correlation with metastasis in sarcoma (* *p* < 0.05).

To investigate the differential expression of ZFP36L2 across various cancer types, we utilized the TCGA database [Figure 1A,B]. Our analysis revealed variations in ZFP36L2 expression by comparing normal tissue samples with corresponding tumor samples, and different tumor types exhibited distinct expression patterns. Subsequently, through TCGA database analysis using the Wilcoxon rank-sum test, we established a significant correlation between ZFP36L2 levels and sarcoma metastasis [*p* < 0.05; Figure 1C].

### 3.2. Functional Enrichment Analysis of Differentially Expressed Genes [DEGs] Revealed That ZFP36L2 Exerts an Influence on Both Tumor Cell and Immune Function

**Figure 2:**
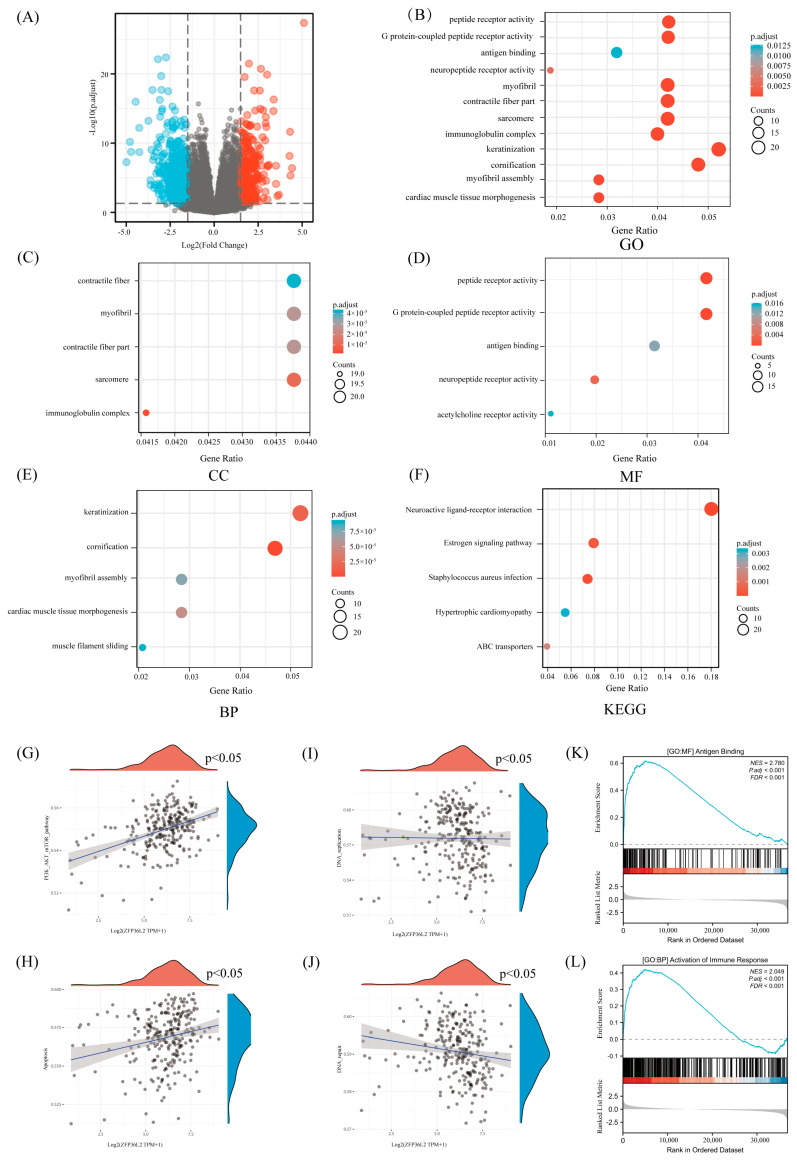
Functional enrichment analysis of the differentially expressed genes [DEGs] between high and low ZFP36L2 expression in sarcoma patients. (**A**) A volcano plot of the differentially expressed genes. (**B**) GO enrichment analysis of DEGs. (**C**) Enriched GO terms in the “cellular component” category. (**D**) Enriched GO terms in the “molecular function” category. (**E**) Enriched GO terms in the “biological process” category. (**F**) KEGG pathway annotations. (**G**–**J**) The correlation between ZFP36L2 and PI3K-AKT-mTOR, apoptosis, P53, DNA replication, and DNA damage repair. (**K**,**L**) The correlation between ZFP36L2 and immunity.

We examined the gene expression profiles of the ZFP36L2 high- and low-expression groups to identify variances in median mRNA expression. A total of 652 differentially expressed genes [DEGs] were derived from the RNA-seq-HTSeq-Counts data [Figure 2A]. Then, we conducted a comprehensive analysis to obtain deep insights into the functional implications of these DEGs and discern the contrast between high and low ZFP36L2 expression levels in SARC. The Gene Ontology [GO] analysis [Figure 2B] results indicated that it is related to cellular and molecular biology, particularly in the context of muscle function, immune response, and cellular differentiation. The same results have been demonstrated for cellular components [CC; Figure 2C], molecular functions [MF; Figure 2D], and biological processes [BP; Figure 2E]. Kyoto Encyclopedia of Genes and Genomes [KEGG] analysis [Figure 2F] is related to muscle structure and function, as well as immune system components. ZFP36L2 exhibited connections with multiple cell proliferation signaling pathways, such as the PI3K-AKT-mTOR pathway [Figure 2G] and the P53 pathway [Supplementary Material Figure S1A]. Additionally, it plays an essential role in the cell cycle encompassing apoptosis [Figure 2H], DNA replication [Figure 2I], and repair [Figure 2J].

We also conducted Gene Set Enrichment Analysis [GSEA] to investigate the biological pathways associated with SARC at varying ZFP36L2 expression levels. The GSEA results indicated that ZFP36L2 is related to immunity. Notably, disparities emerged in the enrichment of pathways within the MSigDB Collection [c5.all.v7.5.1.symbols.gmt]. These findings underscore the connection between ZFP36L2 and immune function [Figure 2K,L; Supplementary Materials Figure S1B–F].

### 3.3. ZFP36L2 Exhibits a Close Association with Macrophages and T Cells

**Figure 3:**
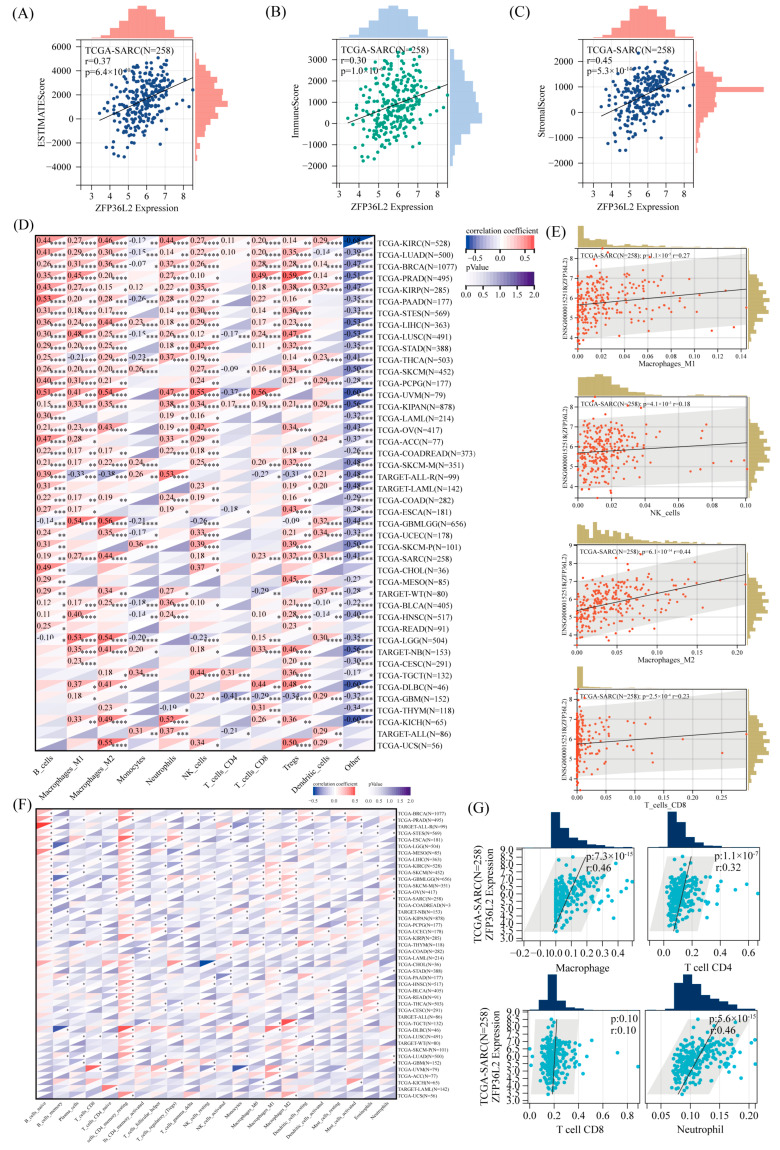
Immune infiltration analysis of ZFP36L2. (**A**–**C**) ZFP36L2 is positively correlated with EstimateScore, ImmuneScore, and StromalScore. (**D**,**E**) The QUANTISEQ algorithm was used to demonstrate ZFP36L2’s correlation with macrophages and CD8^+^ T cells. “*” indicates *p* < 0.05, “**” indicates *p* < 0.01, “***” indicates *p* < 0.001, and “****” indicates *p* < 0.0001. (**F**) CIBERSORT analysis demonstrated a strong association between high ZFP36L2 expression and macrophages, as well as CD4^+^ T cells. (**G**) The TIMER algorithm was used to demonstrate ZFP36L2’s correlation with macrophages and CD8^+^ T cells.

Given the tumor-specific expression patterns of ZFP36L2 across various cancer types, we hypothesized that these differences are linked to variations in the TME, which in turn is intricately tied to the immune response. To estimate the composition of stromal and tumor cells within the TME, we applied the Expression Data [ESTIMATE] algorithm, which provides metrics such as EstimateScore, ImmuneScore, and StromalScore [Figure 3A–C]. Our analysis consistently revealed a positive correlation between ZFP36L2 expression and these three scores. To minimize potential bias, we conducted multiple immune infiltration analyses using QUANTISEQ [Figure 3D,E] and TIMER [Figure 3G]. These analyses affirmed a positive relationship between ZFP36L2 expression and the presence of macrophages and CD8^+^ T cells. Additionally, the CIBERSORT analysis [Figure 3F] demonstrated the strong association of high ZFP36L2 expression with macrophages and CD4^+^ T cells, particularly in sarcomas.

### 3.4. ZFP36L2 Can Be Utilized as a Valuable Prognostic Marker at Elevated IL1β Expression Levels

**Figure 4:**
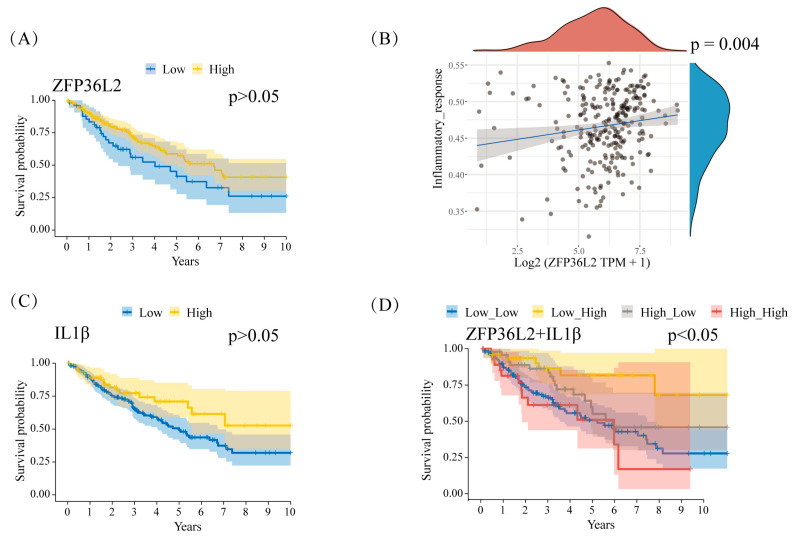
ZFP36L2 is associated with prognosis in IL1β^+^ osteosarcoma patients. (**A**) The KM curve of ZFP36L2 and ten-year survival in osteosarcoma patients. (**B**) The correlation between ZFP36L2 expression and inflammatory response. (**C**) The survival curve of IL1β in the ten-year survival of osteosarcoma patients. (**D**) A KM plot of ZFP36L2 in the ten-year survival rate of IL1β^+^ osteosarcoma patients (*p* < 0.05).

A survival analysis of ZFP36L2 was conducted using data from the TARGET database. The Kaplan–Meier survival curve displayed no statistically significant differences [*p* > 0.05] in overall patient survival [Figure 4A]. Subsequently, we investigated the relationship between ZFP36L2 and inflammatory response [Figure 4B], given that macrophages are known to secrete various inflammatory factors, including IL1β [28]. In addition, we observed that IL1β alone did not exhibit a significant association with survival [*p* > 0.05; Figure 4C]. However, when IL1β expression was elevated, ZFP36L2 emerged as a valuable prognostic indicator, demonstrating statistical significance [*p* < 0.05, adj*p* < 0.05; Figure 4D].

### 3.5. Analysis of Single-Cell Sequencing Data Revealed a Notable Enrichment of ZFP36L2 in CD8^+^ T Cells and IL1β^+^ Macrophages

**Figure 5:**
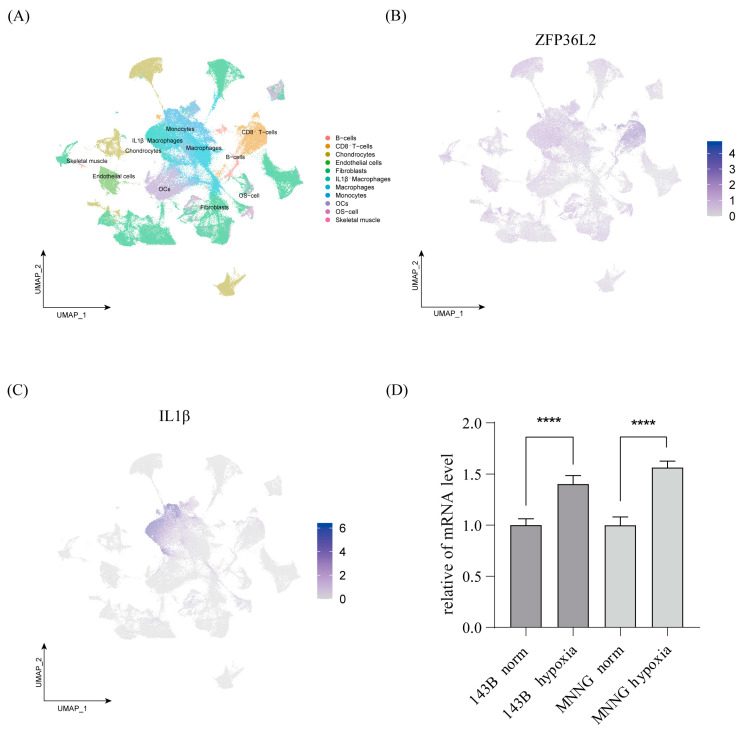
Differential expression in single-cell samples of osteosarcoma. (**A**) Uniform Manifold Approximation and Projection [UMAP] visualization of 11 distinct cell clusters in two OS single-cell datasets. (**B**,**C**) UMAP visualization of the expression levels of ZFP36L2 and IL1β. (**D**) The QPCR findings revealed that under hypoxic conditions, the expression levels of ZFP36L2 are notably elevated in the 143B and MNNG/HOS CL cell lines compared to normoxic conditions (**** *p* < 0.0001).

To further elucidate the association between ZFP36L2 and these immune cell types, we conducted single-cell sequencing analysis. A total of 11 cell subgroups were identified through cell annotation. The umap plot was used to display cell clustering [Figure 5A]. The expression of ZFP36L2 in these cell subpopulations indicated that it is mainly enriched in CD8^+^ T cells and IL1β^+^ macrophages [Figure 5B]. Subsequently, a single-cell sequencing analysis of IL1β within the same database demonstrated the substantial enrichment of IL1β^+^ macrophages [Figure 5C]. qPCR was performed on two OS cell lines [143B and MNNG/HOS CL] with hypoxia and normoxia, and the results showed that the mRNA levels of ZFP36L2 were higher under hypoxia than normoxia.

### 3.6. CellChat Analysis Showed the Interaction Among Tumor Cells, CD8^+^ T Cells, and IL1β^+^ Macrophages

**Figure 6:**
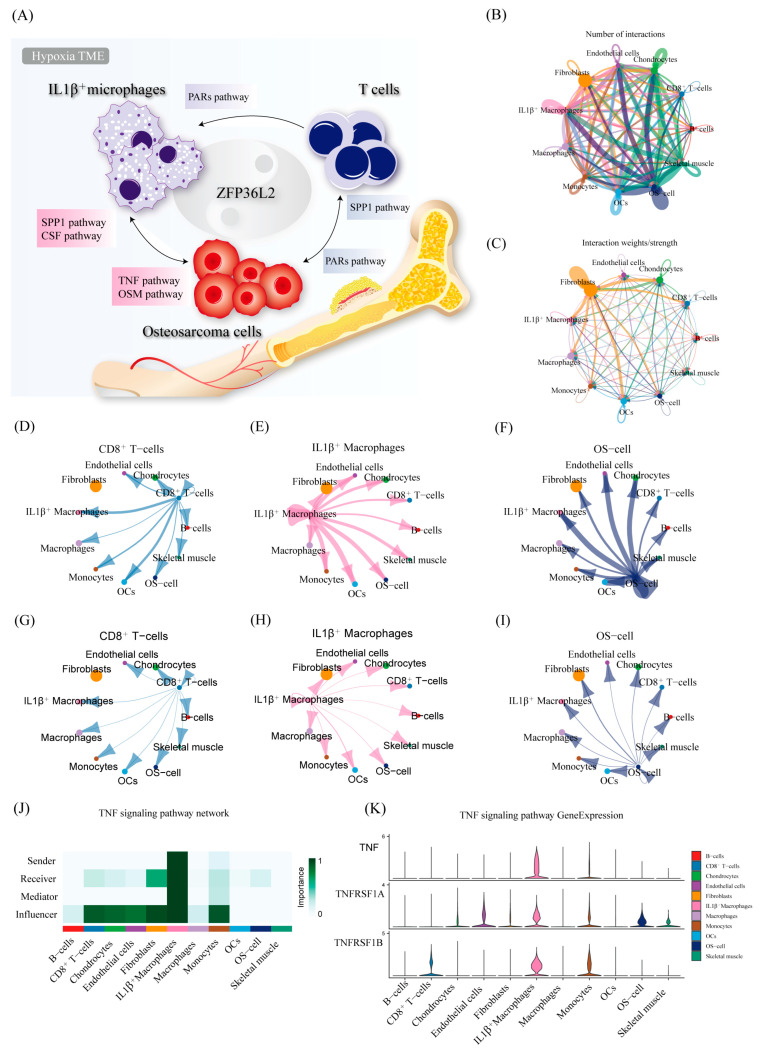
Cellchat analysis of intercellular communication between cell clusters. (**A**) A pattern diagram unveiling the potential ligand–receptor relationships among tumor cells, IL1β^+^ macrophages, and T cells. (**B**,**C**) Circular plots illustrating the number of interactions and the strength of interactions among 11 distinct cell groups. Arrows indicate the direction of intercellular communication. (**D**–**F**) Network plots demonstrating the number of interactions between osteosarcoma [OS] cells, CD8^+^ T cells, and IL1β^+^ macrophages. (**G**–**I**) Network plots illustrating the strength of communication interactions between OS cells, CD8^+^ T cells, and IL1β^+^ macrophages. (**J**) A heatmap displaying the number of potential ligand–receptor pairs in the predicted cell types. (**K**) A violin diagram showing the expression levels of receptor ligands on various types of cells along the TNF pathway.

To comprehensively explore cell–cell interactions, we applied CellChat to study the interrelationships among OS cells, CD8^+^ T cells, and IL1β^+^ macrophages [Figure 6A–C; Supplementary Materials Figure S2]. The quantities [Figure 6D–F] and proportions [Figure 6G,H,I] of the interactions indicated extensive connections. By calculating the Euclidean distance between any pair of shared signaling pathways in the two groups, we found that ligand–receptor pathways play an important role in the mediation and alteration of the TME in addition to intercellular interactions. By studying the interrelationship between tumor cells and immune cells, we found that OS cells affect IL1β^+^ macrophages through the TNF signaling pathway [Figure 6J]. In the TNF pathway, IL1β^+^ macrophages mainly interact with receptors on OS cells and T cells through the ligand TNF [Figure 6K].

## 4. Discussion

ZFP36L2 is an anti-inflammatory protein that regulates posttranscription mRNA decay, playing a critical role in microenvironment immune interactions and tumor cell proliferation. This protein is associated with various inflammatory diseases [29], including multiple sclerosis [30] and systemic lupus erythematosus [31], and mediates the posttranscriptional regulation of immune responses and immune cell function. Notably, ZFP36L2 is a tissue-specific protein [32] that enhances the invasiveness of cancer cells, as observed in pancreatic ductal adenocarcinoma [33]. Its inhibition can reduce cancer cell proliferation [34]. However, our understanding of its function within the TME and its specific role in sarcoma remains limited.

This study highlights the high expression levels of ZFP36L2 in patients with sarcoma and its low expression levels during metastasis. This finding indicates that slight fluctuations in ZFP36L2 can affect tumor behavior, which correlates with clinical metastasis, but not with overall survival. This apparent paradox arises because metastasis is a key adverse factor in sarcoma prognosis that can be overlooked if survival rate alone is considered. These findings suggest that ZFP36L2 is either an upstream candidate in a signaling cascade, or it is expressed in specific cells crucial for tumor metastasis, or both. Nevertheless, each scenario underscores the importance of ZFP36L2 in cancer progression. High levels of ZFP36L2 expression promote cell proliferation in sarcomas. Moreover, associations between ZFP36L2 and key cell proliferation signaling pathways, such as the PI3K–AKT–mTOR and P53 pathways, along with its involvement in cell cycle processes, such as apoptosis, DNA repair, and replication, were uncovered. These results suggest that ZFP36L2 exerts its influence on tumors by disrupting the normal cell cycle. Our results align with the findings obtained from glioblastoma research [34], that is, inhibiting ZFP36L2 expression can promote tumor apoptosis.

Metastasis is a multifaceted process in tumor progression involving tumor and immune cells. The GSEA and GO pathway enrichment analysis demonstrated links between ZFP36L2 and immune function. Given the substantial correlation of ZFP36L2 with metastasis rates in patients with sarcoma and the GSEA results, our investigation extended to the tumor immune microenvironment. We conducted immune infiltration analyses using ESTIMATE, QUANTISEQ, CIBERSORT, and TIMER. Our results showed that ZFP36L2 expression was associated with macrophages and T cells. Macrophages play a crucial role in tumor proliferation and metastasis, and their transition between M1 and M2 phenotypes is influenced by the surrounding environment. M1 macrophages exhibit antitumor activity [35,36,37], but an increase in M2 macrophages can promote tumor progression [38,39,40]. IL1β, which is a cytokine primarily released by M1 macrophages, is considered a prognostic marker in many types of cancer [12,39,41], but not in sarcomas. In numerous cancer types, an increase in M1 macrophages is associated with improved survival and clinical outcomes [42,43]. Therefore, we screened ZFP36L2 and the signature cytokines secreted by M1 macrophages and conducted a Kaplan–Meier survival analysis using the OS database [OS is the most malignant sarcoma]. The results indicated that ZFP36L2 is a prognostic marker when IL1β is highly expressed. The ZFP36L2^low^/IL1β^high^ dual marker is associated with better overall survival and represents a favorable marker for prognosis.

ZFP36L2 demonstrates notable heterogeneity across various cell components in different types of cancer, including OS [44]. Survival analysis further revealed its heterogeneous expression within distinct sarcoma subgroups. To examine cellular heterogeneity in OS and ZFP36L2 expression in specific cells, we conducted single-cell analysis. The single-cell analysis showed that ZFP36L2 was predominantly expressed in the IL1β^+^ macrophages and CD8^+^ T cells, and IL1β was mainly expressed in macrophages [Figure 6], corroborating our initial hypothesis. The simultaneous expression of ZFP36L2 and IL1β in IL1β^+^ macrophages suggests a pivotal role of macrophages in the production of cytotoxic effects against tumor cells and the enhancement of prognosis. Further research is needed to determine how ZFP36L2 functions as a prognostic marker, particularly at elevated IL1β expression levels.

IL1β macrophages accumulate under hypoxic conditions [45]. Consequently, the heightened expression of IL1β suggests a hypoxic TME. Under these conditions, ZFP36L2 plays a pivotal role in the downregulation of T cell-produced cytokines. Consequently, the decreased expression of ZFP36L2 leads to the increased secretion of cytokines, such as IL1β, MMP-9, and TNF-α, by T cells, thereby promoting tumor apoptosis. Moreover, under hypoxic conditions, ZFP36L2 facilitates the recruitment of macrophages. The findings from CellChat analysis revealed that IL1β macrophages and OS cells exert their influence through the TNF pathway, ultimately enhancing antitumor responses.

In this study, we first found that ZFP36L2 has lower expression levels in metastatic sarcoma, but this has no statistical significance for survival. Through further research, we elucidated the prognostic significance of ZFP36L2 in patients with IL1β^+^ OS. This may contribute to inhibiting osteosarcoma metastasis and improving patient survival rates, but certain aspects still need further investigation. While systematically analyzing the ZFP36L2 and IL1β interactions in OS, we encountered challenges in identifying the specific pathways orchestrating TME homeostasis, and patients’ outcomes were affected. Our research results still have limitations, such as the lack of in vitro experiments for validation and the need for a larger cohort to obtain more patient information. We hope that our findings can pave the way for novel treatment modalities in clinical practice.

## 5. Conclusions

This study elucidates the specific expression pattern of ZFP36L2 in tumors, revealing its impact on metastasis and its nonsignificant effect on prognosis. Through GO and KEGG analyses, we uncovered the effects of ZFP36L2 on immunity, prompting an exploration into the TME. Immune infiltration analysis revealed the close association of ZFP36L2 with macrophages and CD8^+^ T cells. Further investigation within the subgroup of OS in sarcoma unveiled a relationship between ZFP36L2 and IL1β, suggesting its potential as a prognostic marker and highlighting the heterogeneity of sarcomas. Regarding cellular heterogeneity, single-cell sequencing analysis showed the expression of ZFP36L2 in IL1β^+^ macrophages, supporting our prognostic findings. Moreover, our research indicates that a decrease in ZFP36L2 level affects a target TNF at high IL1β expression levels, thereby enhancing cytotoxicity and ultimately improving prognosis.

## Data Availability

All data generated or analyzed during this study are included in this published article and its Supplementary Information files.

## Supplementary Materials

The following supporting information can be downloaded at: https://www.mdpi.com/article/10.3390/biomedicines12122861/s1, Figure S1: (A) The correlation between ZFP36L2 and P53 pathway. (B–F) The correlation between ZFP36L2 and immunity; Figure S2: (A–C) The Cellchat analysis revealed lig-and-receptor relationships among tumor cells, IL1β^+^ macrophages, and T cells.
