# Supplementary material for: ZFP36L2 Is a Potential Prognostic Marker of IL1β+ Osteosarcoma
Source: Biomedicines. 2024 Dec 17;12(12):2861. doi: 10.3390/biomedicines12122861 (PMC11673156; doi:10.3390/biomedicines12122861)
Supplement: Supplementary file 1 [file biomedicines-12-02861-s001.zip › Supplementary Materials S1.pdf]

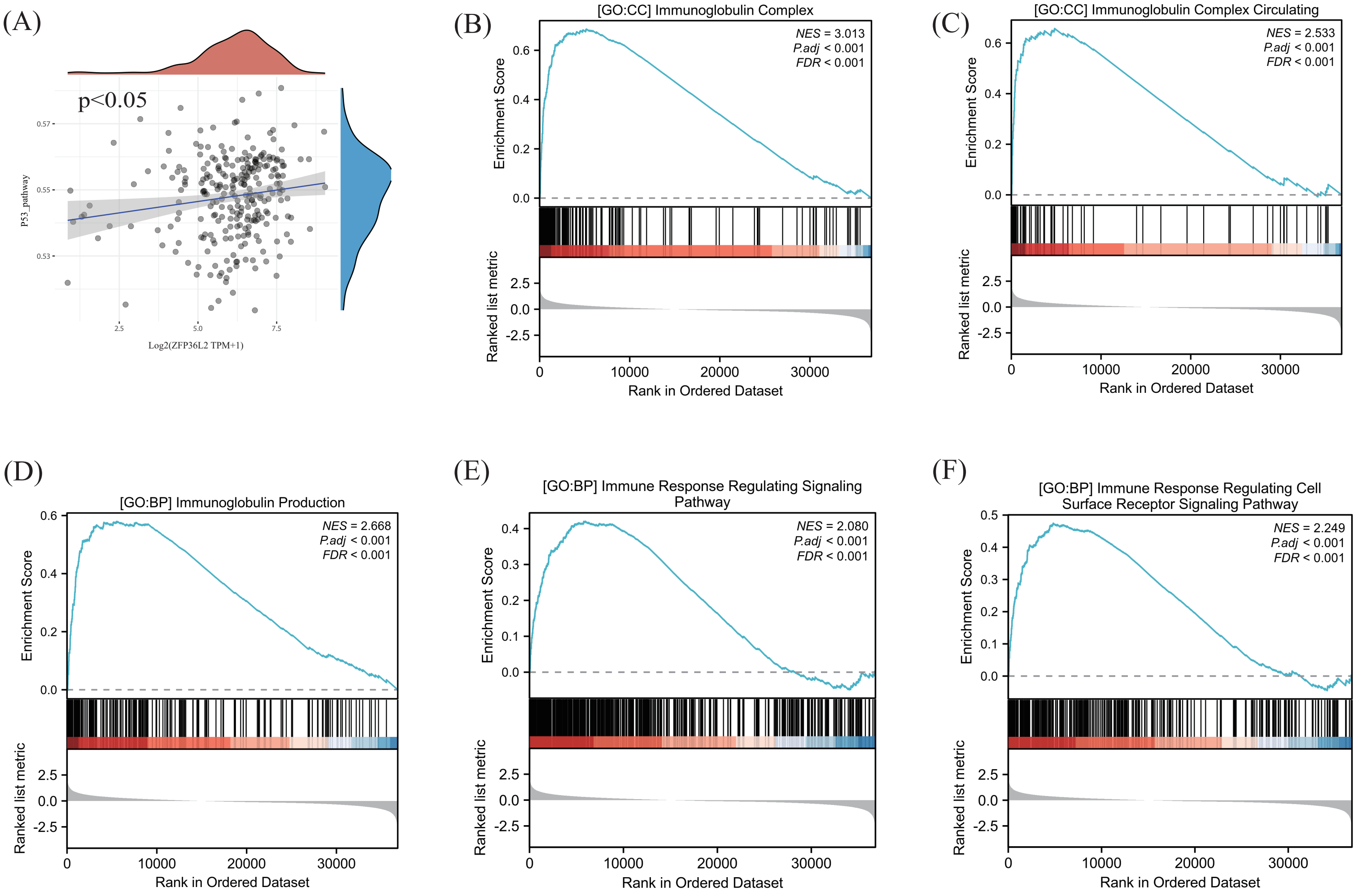

Figure S1. [A] The correlation between ZFP36L2 and P53 pathway. [B, C, D, E, F] The correlation between ZFP36L2 and immunity.
