# Supplementary material for: ZFP36L2 Is a Potential Prognostic Marker of IL1β+ Osteosarcoma
Source: Biomedicines. 2024 Dec 17;12(12):2861. doi: 10.3390/biomedicines12122861 (PMC11673156; doi:10.3390/biomedicines12122861)
Supplement: Supplementary file 1 [file biomedicines-12-02861-s001.zip › Supplementary Materials S2.pdf]

(A)

(B)

(C)

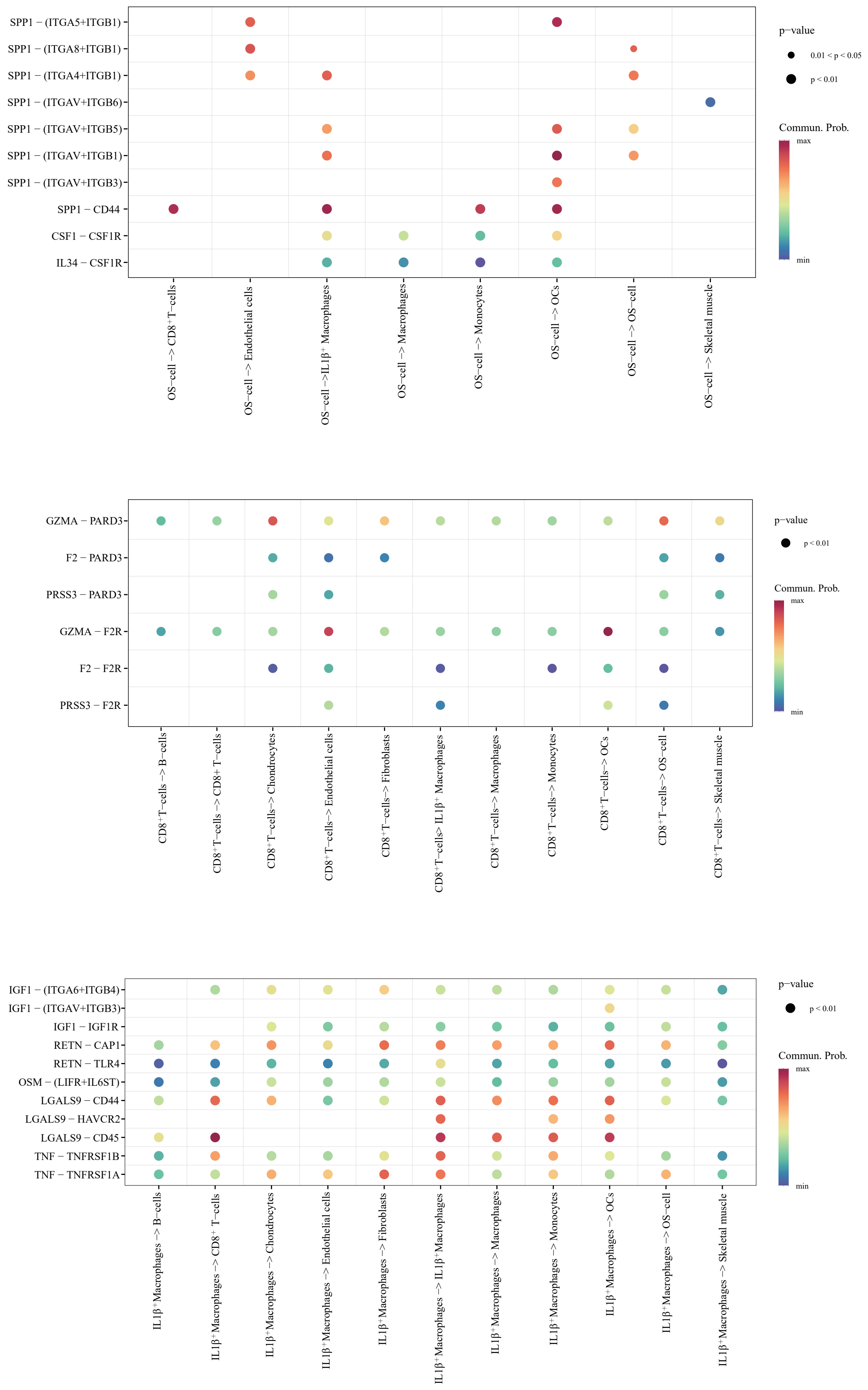

Figure S2. [A, B, C] The Cellchat analysis revealed ligand-receptor relationships among tumor cells, IL1β+ macrophages, and T cells.
